# A Review of Contact Lens-Related Risk Factors and Complications

**DOI:** 10.7759/cureus.30118

**Published:** 2022-10-10

**Authors:** Shrutika V Waghmare, Sandhya Jeria

**Affiliations:** 1 Ophthalmology, Jawaharlal Nehru Medical College, Datta Meghe Institute of Medical Sciences, Wardha, IND

**Keywords:** dry eye, hypoxia, microbial keratitis, ocular allergy, corneal infections, contact lens complications

## Abstract

The purpose of this article is to develop a modern strategy for handling difficulties related to contact lenses and their care. A growing number of people throughout the world are currently concerned by eye-related undesirable activities in allergy sufferers and those wearing contact lenses. While many wearers who experience ocular discomfort exhibit dryness as a symptom, many other contact lens-related pain symptoms also include irritation and fatigue, and managing coexisting diseases must be done in accordance with aspects of wearing contact lenses, all of which undoubtedly increase discomfort.

It is typical for contact lens storage containers to have persistent microbial contamination, which has been linked to microbial keratitis (MK) and clear corneal invasion. Contact lens-associated MK is an interesting, potentially sight-threatening complexity arising from wearing soft contact lenses. Estimates show that for every 10,000 persons who wear contact lenses each year, there are 2 to 5 occurrences of MK. Investigating separate determinants for contact lens-associated MK and evaluating their impact on infection load is one of the challenges in their administration. It is hoped that this will offer a useful outline of the complicated issues of contact lens wear that are both infectious and non-infectious. Recent epidemiological studies detailing the risk factors associated with contact lens use, and the effect of pathogen and individual immune profiles on the severity of diseases have enlightened how we might interpret the prophylaxis and prevention of contact lens-related corneal infection. The most dreaded side effect of contact lens use, infectious keratitis linked with contact lenses, will be reviewed, along with the most recent advancements in its diagnosis and treatment.

## Introduction and background

**Figure 1:**
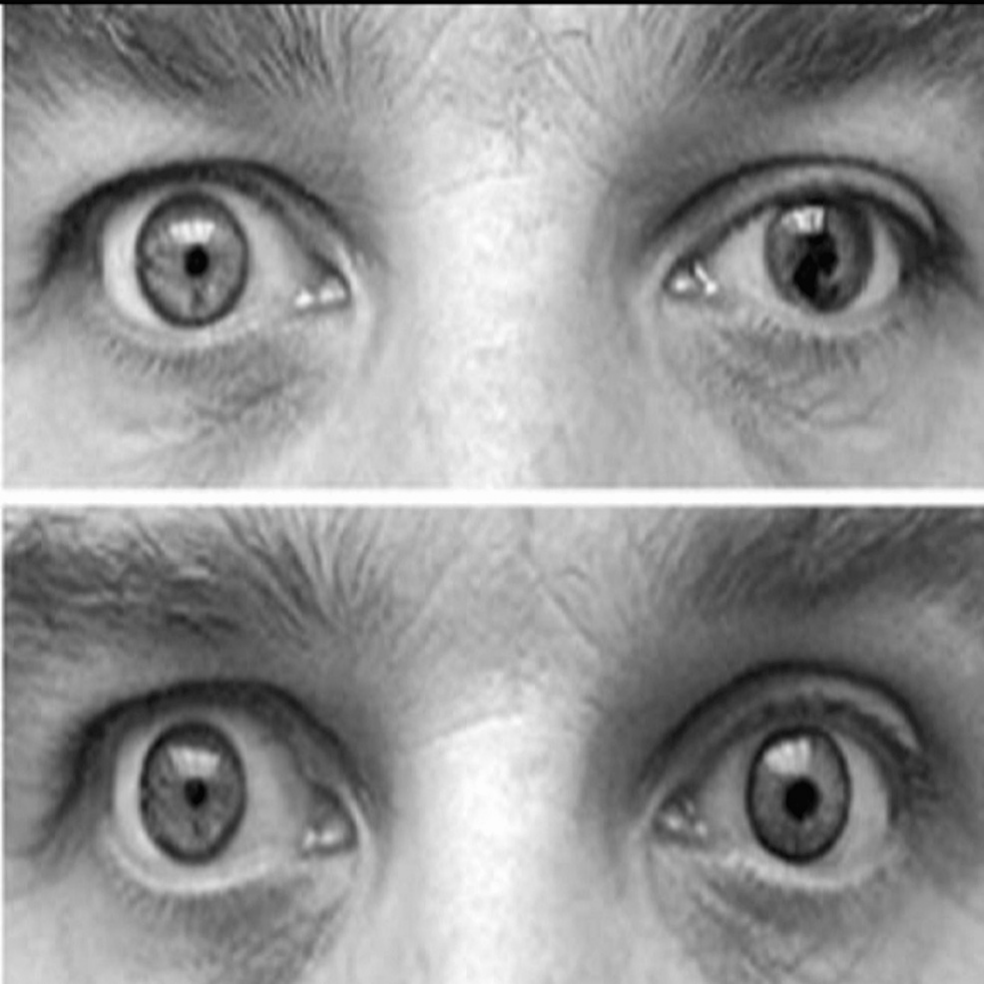
Using Prosthetic Lenses to Achieve a More Natural Appearance in a Patient With Iris Coloboma. This figure has been taken from open access journal under a CC-BY license source [2].

**Figure 2:**
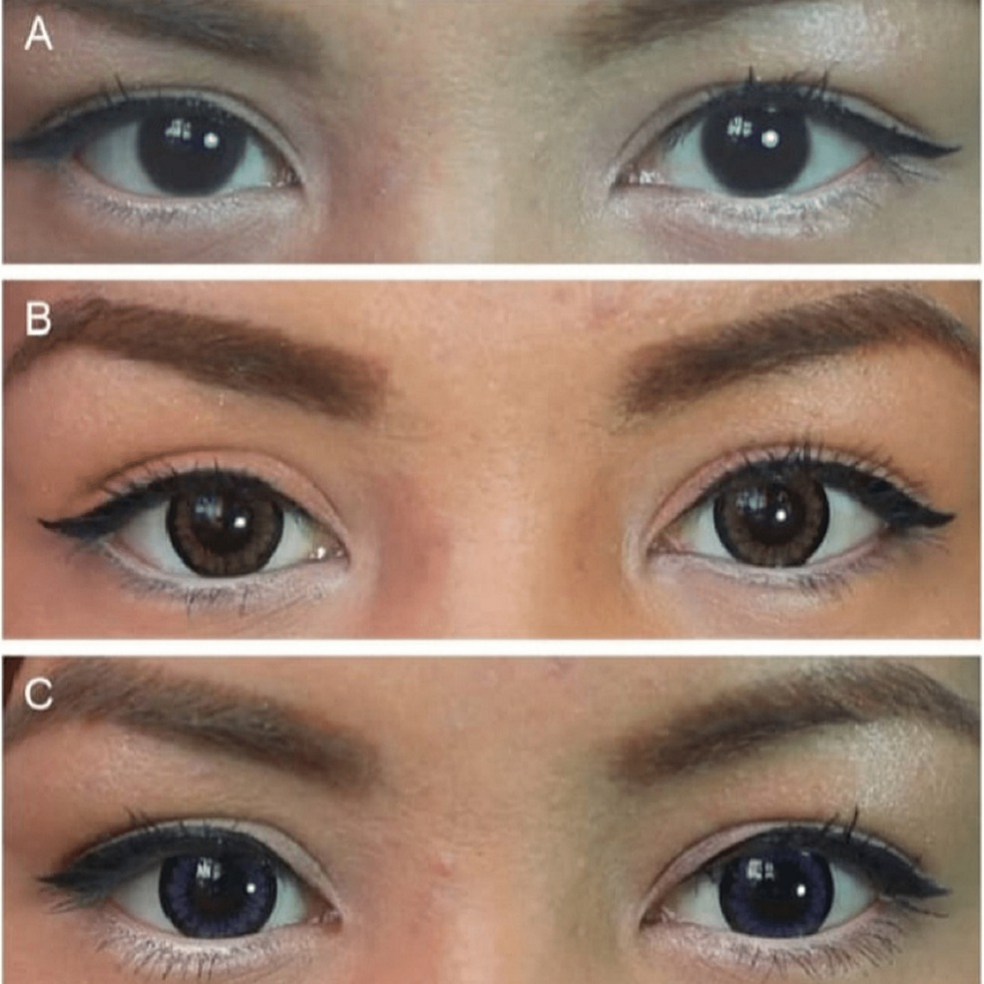
Utilization of (a) Panel, Exhibit the Appearance Preceding to Lens Wear. (b) Brown and (c) Blue Cosmetic Contact Lenses to Accomplish a Varied Cosmetic Impact on an Individual. This figure has been taken from open access journal under a CC-BY license source [2].

Contact lens is a common artificial device used to correct refractive error, whose front surface substitutes the foremost surface of the cornea. Wearing contact lenses is becoming more and more common, with a growing number of indications, including refractive error repair, controlling nearsightedness, restorative reasons, and cosmetic uses [1,2]. Cosmetic contact lenses, also known as circular or decorative lenses, were initially developed for patients with iris and corneal distorting defects Figure 1, but they now also change or enhance a person's appearance (Figure 2) [2].

The following three types of contact lenses hard lenses, rigid gas-permeable lenses, and soft lenses can be distinguished based on the concept of the material used in their production. In several countries, the prevalence of contact lens uses by adult groups aged 15 to 25 years was 40.5%, with females being the predominant group affected [3]. Approximately there are 125 million contact lens wearers globally, with a 6% increase annually based on sales growth [3,4]. As long as patients wear contact lenses continuously to correct their refractive error, addressing unhygienic practices, overnight use, and contact lens solution-induced complications will remain a point of concern in ophthalmic practice [4]. The increased oxygen permeability of contact lenses has been related to decreased initial microbial binding to corneal cells and increased exfoliation of superficial epithelial cells, but MK prevention has not been shown [5,6]. MK is a feared complication in contact lens wearers and it is estimated that nearly one in 500 wearers per year will develop MK with overnight use of contact lenses [6]. Risk factors that have always been acknowledged might be categorized as either modifiable or non-modifiable risk factors. Across many climates and cultures, there are variations in lens supply patterns, wearer behavior, contact lens prescriptions, and natural microbiota. These studies also outline risk factors for microbial keratitis linked to contact lenses and assess their impact on disease load [7].

## Review

Epidemiology

The highest occurrence of infectious keratitis is caused by an overnight soft contact lens with a prevalence of 9.2 to 20.9 per 10,000 users. This is followed by a daily soft contact lens with a prevalence of 2.2 to 4.5 and a rigid gas-permeable contact lens with a prevalence of 0.4 to 4.0 per 10,000 users [8]. The risk of MK is lowest in daily disposable contact lenses. The prevalence of MK is one in every 500 users for both short-term silicone hydrogel and hydrogel contact lenses [9]. 

Etiology

Depending on the environment, different causal organisms can cause different types of infectious keratitis. In general, the frequency of the causative microorganisms of contact lens-associated infectious keratitis is revealed (Table 1), along with gram-positive bacteria isolated from temperate climates, fungi, and gram-negative bacteria recovered in sub-tropical and tropical climates [8-10].

**Table 1:** The Prevalence of the Agents Responsible for Infectious Keratitis Linked to Contact Lenses. This table has been taken from open access journal under a CC-BY license [8-10].

Microorganism	Frequency (%)
Pseudomonas aeruginosa	6-55.55%
Other coagulase-negative staphylococcus spp.	8-17.64%
Serratia marcescens	2-17.1%
Staphylococcus aureus	2-12.5%
Acanthamoeba spp.	1.96-12.5%
Fusarium spp.	2-12.5%
Propionibacterium acnes	11.76%
Mycobacterium chelonae	6.4%
Streptococcus spp.	3.92-5.9%
Nocardia spp.	1-1.96%
Klebsiella	0-1%

Risk factors 

Among the various risk factors for contact lens-induced infectious keratitis, the two most frequent ones are poor hygiene and overnight wear, which are responsible for 33% and 43% of the cases, respectively [8]. It was discovered that extended wear and unskilled wearers increase the risk of corneal infection during overnight wear. In severe keratitis, lack of replacement and poor hygiene (mishandling of contact lenses) result in 63% of the population's associated risk for fungal and bacterial infection [11,12]. Also, swimming, showering, or washing contact lenses in freshwater are risk factors for Acanthamoeba keratitis, and traveling is related to routine wearing changes [12] (Table 2).

**Table 2:** Modifiable and Non-modifiable Risk Factors Related to Contact Lens-associated Infectious Keratitis. This table has been taken from open access journal under a CC-BY license [12].

Contact Lens-associated Infectious Keratitis	
Modifiable Risk Factors	Non-modifiable Risk Factors
Wear schedule	Gender
Days of weekly use	Age
Contact lens type	Socioeconomic status
Hand washing before cleaning	Previous ocular trauma
Poor lens hygiene	Genetic predisposition
Current smoker	Hypermetropia (as compared to myopia)
Case hygiene/replace time	
Water exposure, including showering with contact lenses	

Complications

Hypoxia-Induced Changes

The appearance of profoundly oxygen-penetrable silicone hydrogel contact lenses being oxygen permeable, a decline in the frequency of hypoxic complexities [13]. Corneal oxygen requirements also differ among individuals. The avascular cornea derived oxygen directly through the tear film. Cornea also depends on pre-corneal tear film for nutrients (glucose and others) and carrying away waste products. Therefore, overwear of contact lenses disrupts this process. Previously reported nonreversible complications include polymegethism, corneal vascularization, and pleomorphism [9,13]. Rigid gas permeable lenses and silicone hydrogel contact lenses should be advised to decrease complications of hypoxia [14].

Inflammatory Conditions

There are many different clinical presentations, ranging from the presence of asymptomatic, isolated to diffuse infiltrates linked to the inflammatory response [2]. Recognized risk factors for corneal infiltrative events related to contact lenses include lens material, age, wear modality, hygiene practice, duration of wear, and overnight wear [15]. The prevalence of noninfectious inflammatory events associated with contact lenses varies with the material of the lens and lens replacement schedules. In contrast to controls, patients who had the contact lens-associated acute eye response (CLARE), an inflammatory reaction of the cornea and conjunctival related to eye closure and prolonged lens wear regimens, had a bulbar and limbal conjunctival injection and conjunctival staining [2,16].

Allergic and Toxic Reactions

There are numerous materials and contact lens solutions on the market, each with a different formulation and composition. To help clean, lubricate, and disinfect lenses, multifunction solutions combine buffers, surfactants, preservatives, lubricants, and antimicrobial agents. Approximately 15%-20% of the population have a history of allergies and in 40%-60% of patients, ocular symptoms are present [1,2]. When allergic reactions occur, contact lens wearers are frequently obliged to stop wearing the lenses. Also for cessation of contact lens wear, the anti-allergic eye drops must be introduced several times during the day. Thimerosal and chlorhexidine are examples of low-molecular-weight preservatives that have historically been known to cause hazardous and allergic reactions if ingested. A Patient may present with a range of clinical signs and symptoms. Symptoms may include pain, tearing, contact lens tolerance, and photophobia and signs, including the transition from squamous epithelium of the cornea to conjunctival epithelium near the involved limbus and progressive fluorescein corneal staining [17].

Mechanical Conditions

The normal lid-cornea resurfacing mechanism is disrupted by the contact lens, which behaves as a foreign body and applies mechanical effects to the ocular surface [18]. The impact of contact lens wear on corneal sensitivity is demonstrated by etiological factors, such as hypoxic and mechanical factors [19]. Patients may develop either limbal or paralimbic lesions, also referred to as superior epithelial arcuate lesions (SEAL), located superiorly on the cornea. In the superior fornix, the lenses may also be embedded and dislodged [20]. If not treated for prolonged periods, this can bring about cystic lesions and the formation of foreign body granuloma. The symptoms include the existence of an orbital mass and eyelid retraction or drooping [21]. Additionally, mechanical forces like rubbing, incorrect lens insertion and removal, trauma, and foreign objects wedged between the corneal surface and lens can cause corneal erosion [22]. The ocular surface can be traumatized by the sharp edges of lenses due to any damage to contact lenses. By carefully considering the severity of the disruption of contact lenses, eyecare professionals should administer proper doses of prophylactic antibiotics [23].

Contact Lens-Related Papillary Conjunctivitis

The use of soft lenses, especially silicone hydrogel lenses, has been a risk factor for contact lens-associated papillary conjunctivitis. It is classified as a local and generalized condition [24]. Usual findings are associated with pruritus, erythema, upper palpebral conjunctiva, and mucoid discharge [25]. The wearer may appear to have decreased lens tolerance, whereas essential information can be obtained by checking lens movement and fitness, with increased lens movement identified by examination. Antihistamines, mast cell stabilizers, or drugs used in combination, such as in the treatment of hypersensitive conjunctivitis, could also be considered. Other treatments, such as topical steroids, may be employed [26,27].

Contact Lens-Induced Discomfort and Dryness

The most important factor in people stopping their use of contact lenses is discomfort, which is a persistently difficult problem. Discomfort may influence the discontinuation of lens wear, and lens wearing time decreases, despite advances in new technology and increased knowledge about lens design, fitting, and material [28]. Many wearers who encounter ocular discomfort report that dryness is a common symptom. Other signs include fatigue and irrigation [29]. Wearing contact lenses is a known risk factor for dry eye, with approximately 50% of wearers experiencing dry eye symptoms and possibly indicating dry eye infection [10,30]. Control of dry eye conditions through treatment may include lubricants with supplementation of tears, lipid supplements, and the use of long-chain essential fatty acids in dietary supplementation, lid hygiene, eyelid warming apparatus, and surgical treatment in severe cases [31]. 

Infectious

*Microbial keratitis: *Intriguing but fearful, contact lens-related microbial keratitis is a side effect of wearing contact lenses. The most well-known significant risk factor for microbial keratitis is using contact lenses. An 80-fold increased risk of developing MK in healthy people has been noted. The rate of microbial keratitis in daily wear is estimated to be 2 to 5 people per 10,000 lens wearers per year [6]. Delay of evaluation and initiation of appropriate antibacterial treatment leads to increased morbidity and can contribute to disadvantageous results [32]. The prevalence of bacterial keratitis associated with contact lenses is anticipated to decline with the introduction of silicone hydrogel lenses, which have better oxygen penetrability. It is noteworthy to notice that patients who used daily disposable contact lenses had lower rates of severe illness and a reduced likelihood of losing their vision compared to individuals who used them on alternate days of the week. According to this data, cleanliness may play a significant role in the frequency and severity of contact lens-associated microbial keratitis. Behavioral and demographic risk factors are other risk factors for MK associated to contact lenses. Such as poor hand hygiene, sleeping overnight in contact lenses, every topping of disinfecting solution, and smoking [6,33]. Patients with MK typically present with pain, diminished visual acuity, and photophobia. The patient may also present with eye redness, purulent discharge, eyelid swelling, and excessive tearing. Variation in the incidence of MK depends on climatic circumstances, more likely to occur in warmer and humid weather [34]. Patients with suspected contact lens-related MK should be advised to stop wearing lenses until the resolution of the staining [35]. One of the challenges of microbial keratitis is when to administer corticosteroids. Corticosteroids are introduced when the infection is responding to antimicrobial therapy otherwise it can cause an increase in severity. The patients who presented with central ulceration and poor visual acuity also benefit from initial corticosteroid therapy [36]. Early diagnosis and focused remedy prevent rapid destruction of the eye. Corneal scraping to gain epithelial samples or confocal microscopy is mandatory in all suspected corneal infections. Pseudomonas aeruginosa is the most frequent causative agent of contact lens-related keratitis and is related to drastically worse effects than in other bacterial infections [5]. Immoderate proteolytic activity by pseudomonas aeruginosa mediated through its enzymes and toxins can damage the cornea. Amoebic keratitis is an uncommon entity and about 90% of cases are related to contact lenses [9]. Their use can cause microtrauma in the corneal epithelium, and when mixed with the contaminated surface of contact lenses it becomes a primary chance factor.

Keratitis is caused by Acanthamoeba, a free-living single-celled protist. It is a dangerous yet uncommon condition that can lead to blindness and is challenging to cure. The pathophysiology of this living organism is well understood, although now not properly perceived. Contact lens wear, on the other hand, has been identified as a significant hazard issue and typically entails the lenses being exposed to contaminated water sources [37,38]. Specific risk factors include lenses stored in water, handling lenses with wet hands, and flushing cases in running water before lens storage. Long-term usage of biguanide- or diamindine-based antimicrobial medications, which have potent cysticidal effects, has been advised due to the amoebic cysts' resilience to therapy [1,39]. Other remedy preferences that in most cases have been reported usually include cryotherapy, crosslinking, and epithelial debridement [40]. 

## Conclusions

Safe contact lenses are an effective form of vision correction for the millions of people who require them; however, they are not devoid of risks. The risk of eye infection increases due to a lack of care and personal cleanliness such as topping off storage cases with disinfection solution and washing contact lenses in fresh water. It is uncommon in this demographic to use contacts for prolonged periods or a greater variety of activities. Since contact lens technology has advanced, individuals who prefer not to wear glasses frequently select this method of vision correction. To be able to intervene as necessary, eye care practitioners must be fully aware of the variety of potential problems that lens wearers may encounter. This will motivate our patients to use this secure and effective method of vision restoration. Poor hand hygiene, wearing contacts accidentally at night, and the method of lens maintenance are all free risk factors for microbial keratitis linked to contact lenses. Prevention efforts could include vigorous health promotion activities that increased awareness about the importance of proper contact lens hygiene that can encourage contact lens wearers to adopt healthy habits, such as keeping all water away from contact lenses, discarding used disinfecting solutions from the case, and cleaning with fresh solution each day, and replacing their contact lens case every three months, can reduce their chances of getting an eye infection.

## References

[1] Lim CH, Stapleton F, Mehta JS (2018). Review of contact lens-related complications. Eye Contact Lens.

[2] Lim CH, Stapleton F, Mehta JS (2019). A review of cosmetic contact lens infections. Eye (Lond).

[3] Zainodin E, Najmee N, Hamzah F, Saliman N (2021). Ocular complications in contact lens wear and the risk factors: A retrospective analysis. Environ-Behav Proc J.

[4] Forister JF, Forister EF, Yeung KK, Ye P, Chung MY, Tsui A, Weissman BA (2009). Prevalence of contact lens-related complications: UCLA contact lens study. Eye Contact Lens.

[5] Ladage PM, Yamamoto K, Ren DH, Li L, Jester JV, Petroll WM, Cavanagh HD (2001). Effects of rigid and soft contact lens daily wear on corneal epithelium, tear lactate dehydrogenase, and bacterial binding to exfoliated epithelial cells. Ophthalmology.

[6] Stapleton F, Keay L, Edwards K, Naduvilath T, Dart JK, Brian G, Holden BA (2008). The incidence of contact lens-related microbial keratitis in Australia. Ophthalmology.

[7] Lim CH, Carnt NA, Farook M, Lam J, Tan DT, Mehta JS, Stapleton F (2016). Risk factors for contact lens-related microbial keratitis in Singapore. Eye (Lond).

[8] Alamillo-Velazquez J, Ruiz-Lozano RE, Hernandez-Camarena JC, Rodriguez-Garcia A (2021). Contact lens-associated infectious keratitis: Update on diagnosis and therapy. IntechOpen.

[9] Stapleton F, Carnt N (2012). Contact lens-related microbial keratitis: how have epidemiology and genetics helped us with pathogenesis and prophylaxis. Eye (Lond).

[10] Kaye R, Kaye A, Sueke H, Neal T, Winstanley C, Horsburgh M, Kaye S (2013). Recurrent bacterial keratitis. Invest Ophthalmol Vis Sci.

[11] Edwards K, Keay L, Naduvilath T, Snibson G, Taylor H, Stapleton F (2009). Characteristics of and risk factors for contact lens-related microbial keratitis in a tertiary referral hospital. Eye (Lond).

[12] Stellwagen A, MacGregor C, Kung R, Konstantopoulos A, Hossain P (2020). Personal hygiene risk factors for contact lens-related microbial keratitis. BMJ Open Ophthalmol.

[13] Stapleton F, Dart J, Minassian D (1992). Nonulcerative complications of contact lens wear. Relative risks for different lens types. Arch Ophthalmol.

[14] Sweeney DF (2013). Have silicone hydrogel lenses eliminated hypoxia?. Eye Contact Lens.

[15] Chalmers RL, Wagner H, Mitchell GL (2011). Age and other risk factors for corneal infiltrative and inflammatory events in young soft contact lens wearers from the Contact Lens Assessment in Youth (CLAY) study. Invest Ophthalmol Vis Sci.

[16] Stapleton F, Ramachandran L, Sweeney DF, Rao G, Holden BA (2003). Altered conjunctival response after contact lens-related corneal inflammation. Cornea.

[17] Dua HS, Saini JS, Azuara-Blanco A, Gupta P (2000). Limbal stem cell deficiency: concept, aetiology, clinical presentation, diagnosis and management. Indian J Ophthalmol.

[18] Alghamdi WM, Markoulli M, Holden BA, Papas EB (2016). Impact of duration of contact lens wear on the structure and function of the meibomian glands. Ophthalmic Physiol Opt.

[19] Lum E, Golebiowski B, Gunn R, Babhoota M, Swarbrick H (2013). Corneal sensitivity with contact lenses of different mechanical properties. Optom Vis Sci.

[20] Moon JY, Park H, Lee JW, Kim SJ (2014). A case of a rigid gas permeable (RGP) contact lens found as a foreign body under the upper tarsal conjunctiva. J Korean Ophthalmol Soc.

[21] Jones D, Livesey S, Wilkins P (1987). Hard contact lens migration into the upper lid: an unexpected lid lump. Br J Ophthalmol.

[22] Ammer R (2016). Effect of contact lens wear on cornea. Pak J Ophthalmol.

[23] Hood CT (2009). The Wills eye manual: office and emergency room diagnosis and treatment of eye disease. Br J Ophthalmol.

[24] Tagliaferri A, Love TE, Szczotka-Flynn LB (2014). Risk factors for contact lens-induced papillary conjunctivitis associated with silicone hydrogel contact lens wear. Eye Contact Lens.

[25] Skotnitsky CC, Naduvilath TJ, Sweeney DF, Sankaridurg PR (2006). Two presentations of contact lens-induced papillary conjunctivitis (CLPC) in hydrogel lens wear: local and general. Optom Vis Sci.

[26] Sorbara L, Jones L, Williams-Lyn D (2009). Contact lens induced papillary conjunctivitis with silicone hydrogel lenses. Cont Lens Anterior Eye.

[27] Anderson DF (2001). Management of seasonal allergic conjunctivitis (SAC): current therapeutic strategies. Clin Exp Allergy.

[28] Dumbleton K, Woods CA, Jones LW, Fonn D (2013). The impact of contemporary contact lenses on contact lens discontinuation. Eye Contact Lens.

[29] Nichols JJ, Willcox MD, Bron AJ (2013). The TFOS international workshop on contact lens discomfort: Executive summary. Invest Ophthalmol Vis Sci.

[30] Nichols JJ, Sinnott LT (2006). Tear film, contact lens, and patient-related factors associated with contact lens-related dry eye. Invest Ophthalmol Vis Sci.

[31] Barabino S, Rolando M, Camicione P, Chen W, Calabria G (2005). Effects of a 0.9% sodium chloride ophthalmic solution on the ocular surface of symptomatic contact lens wearers. Can J Ophthalmol J Can Ophtalmol.

[32] Keay L, Edwards K, Naduvilath T, Taylor HR, Snibson GR, Forde K, Stapleton F (2006). Microbial keratitis predisposing factors and morbidity. Ophthalmology.

[33] Steinemann TL, Pinninti U, Szczotka LB, Eiferman RA, Price FW (2003). Ocular complications associated with the use of cosmetic contact lenses from unlicensed vendors. Eye Contact Lens.

[34] Stapleton F, Keay LJ, Sanfilippo PG, Katiyar S, Edwards KP, Naduvilath T (2007). Relationship between climate, disease severity, and causative organism for contact lens-associated microbial keratitis in Australia. Am J Ophthalmol.

[35] Vijay AK, Willcox M, Zhu H, Stapleton F (2015). Contact lens storage case hygiene practice and storage case contamination. Eye Contact Lens.

[36] Srinivasan M, Mascarenhas J, Rajaraman R (2012). Corticosteroids for bacterial keratitis: the Steroids for Corneal Ulcers Trial (SCUT). Arch Ophthalmol.

[37] Por YM, Mehta JS, Chua JL (2009). Acanthamoeba keratitis associated with contact lens wear in Singapore. Am J Ophthalmol.

[38] Joslin CE, Tu EY, Shoff ME (2007). The association of contact lens solution use and Acanthamoeba keratitis. Am J Ophthalmol.

[39] Elder MJ, Kilvington S, Dart JK (1994). A clinicopathologic study of in vitro sensitivity testing and Acanthamoeba keratitis. Invest Ophthalmol Vis Sci.

[40] Allan BD, Dart JK (1995). Strategies for the management of microbial keratitis. Br J Ophthalmol.

